# Environmental exposure to xenoestrogens and oestrogen related cancers: reproductive system, breast, lung, kidney, pancreas, and brain

**DOI:** 10.1186/1476-069X-11-S1-S8

**Published:** 2012-06-28

**Authors:** Aleksandra Fucic, Marija Gamulin, Zeljko Ferencic, Jelena Katic, Martin Krayer von Krauss, Alena Bartonova, Domenico F Merlo

**Affiliations:** 1 Institute for Medical Research and Occupational Health, Zagreb, Ksaverska c 2, Croatia; 2 University Hospital “Zagreb”, Zagreb, Kispaticeva 12, Croatia; 3 Children’s Hospital “Srebrnjak”, Zagreb, Srebrnjak 100, Croatia; 4 WHO, Regional Office for Europe, Copenhagen, Scherfigsvej 8, Denmark; 5 NILU – Norwegian Institute for Air Research, Kjeller, Norway; 6 National Institute for Cancer Research, Genoa, Largo R. Benzi 10, Italy

## Abstract

The role of steroids in carcinogenesis has become a major concern in environmental protection, biomonitoring, and clinical research. Although historically oestrogen has been related to development of reproductive system, research over the last decade has confirmed its crucial role in the development and homeostasis of other organ systems. As a number of anthropogenic agents are xenoestrogens, environmental health research has focused on oestrogen receptor level disturbances and of aromatase polymorphisms. Oestrogen and xenoestrogens mediate critical points in carcinogenesis by binding to oestrogen receptors, whose distribution is age-, gender-, and tissue-specific. This review brings data about cancer types whose eatiology may be found in environmental exposure to xenoestrogens. Cancer types that have been well documented in literature to be related with environmental exposure include the reproductive system, breast, lung, kidney, pancreas, and brain. The results of our data mining show (a) a significant correlation between exposure to xenoestrogens and increased, gender-related, cancer risk and (b) a need to re-evaluate agents so far defined as endocrine disruptors, as they are also key molecules in carcinogenesis. This revision may be used to further research of cancer aetiology and to improvement of related legislation. Investigation of cancers caused by xenoestrogens may elucidate yet unknown mechanisms also valuable for oncology and the development of new therapies.

## Background

Despite the considerable efforts to decrease environmental pollution we still witness uncontrolled introduction of new compounds in living and working environment. Additionally, pollution control in low income and developing countries has seen limited success. The balance between needs of a fast growing human population and technology/science development is questionable, partially as a consequence that the available knowledge is not always applied in an efficient way as it should be.

The last century's paradigm “one agent - one disease” has helped to identify the major causal pathways and the identification of pollution related diseases, including cancer. Based on this approach, epidemiological studies set off many activities to reduce pollution and prevent exposure. However, a large body of data accumulated over the last decade, with a recent significant contribution of molecular biology, clearly shows that this historical simplistic interpretation of biomonitoring data fails to answer a number of questions about environmental threats to human health.

Cancer incidence monitoring in developed countries is relatively accurate. A better classification of cancer types, the networking of cancer registries, and the increasing population coverage for cancer registration are unfortunately accompanied**,** due to unsolved technical and organizational difficulties, by publishing of cancer register reports with a lag of several years. This lag is a serious obstacle in identifying current environmental health risks and setting timely effective preventive measures.

According to recent data [1], childhood cancer incidence increases 1% a year all over Europe. In the adult population a rising trend is reported for soft tissue sarcoma, brain tumours, germ-cell tumours, lymphomas, renal cancers, leukemias, breast cancer, and lung cancer in women. Breast, colorectal, prostate, and lung cancer are the most commonly diagnosed cancers in the European population [2]. Only limited part of the detected increase may be related to screening programs.

During the last decade, environmental health and oncology have shown an increasing interest in oestrogen as an evolutionary conserved molecule. With its endocrine, paracrine, and neurotransmitting activity [3-5], oestrogen is not limited to the development and regulation of the reproductive system. The distribution of oestrogen receptors in mammalian tissues suggests that oestrogens could have a significant role in orchestrating a number of pathways in living organisms during development and adulthood. Additionally, new evidences confirm a strong impact of this molecule on carcinogenesis [6-9].

Very little is known about changes in oestrogen levels and the tissue ratio between alpha and beta oestrogen receptors (ER) during development [10]. In the second trimester of human foetal development the highest concentrations of ER beta mRNA are found in the testis and the ovary and of ER alpha mRNA in the uterus. Relatively high concentrations of either receptor are also present in the spleen, while low levels are detected in the kidney, thymus, skin, and lung [11]. The pre-pubertal ratio between ERs alpha and beta in human tissues in males and females is not known**.** Additionally, ER alpha and beta are polymorphically distributed and as such they play different roles in carcinogenesis [12-14].

At higher levels, oestrogen is carcinogenic [15]; similar to ionizing radiation it may produce reactive oxygen species and cause hypomethylation and microsatellite instability [9,16-18]. Its metabolites, quinones, cause the formation of DNA adducts, depurination, lipid-derived aldehyde-DNA adducts, and aneuploidy [15,19]. By decreasing glutathione-S-transferases, oestrogens may increase cellular oxidative DNA damage in oestrogen-responsive tissues, when the organism is simultaneously exposed to genotoxicants. This is an early step in the process of carcinogenesis [20].

Gender differences in the incidence of cancers such as the lung, kidney, or pancreas cancer suggest that hormones may play a role in their aetiology [21]. Recent findings suggest that all neoplastic mammalian tissues are characterized by disturbances in ER levels [6]. As gender related estrogen levels in foetal, and prepubertal tissues, the tissue specific ER distribution and oestrogen bimodal activity modulate the development of biological pathways and organogenesis [22,23] some cancer types may have origin in their prenatal and postnatal disturbance caused by exposure to xenoestrogens.

The general population is exposed to a number of hormonally active compounds on a daily basis. These compounds were introduced in the living environment during the last few decades, the majority of which are xenoestrogens. Chemicals like polycyclic aromatic hydrocarbons (PAH), pesticides, polychlorinated biphenyl (PCB), dichlorodiphenyl-trichlorethane (DDT), some drugs (e.g., antiepileptic drugs), fungicides, cotinine, phytoestrogens, mycotoxins, bisphenol A (a plastics additive), phthalates, alkylphenols, and metalloestrogens mimic oestrogen action, affect oestrogen levels, or bind to oestrogen receptors [24-29]. Xenoestrogens are present in a number of substrates such as cigarette smoke, automobile exhaust, chemical industry pollutants, grilled meat, volcano dust, forest fire smoke, milk, water, and cosmetic products. This means that all human population may be exposed to them.

This article seeks to give an insight in how environmental exposure to xenoestrogens relates to breast, lung, kidney, brain, pancreas, and reproductive system cancers, which are all characterized by disturbances in ER.

## Breast cancer

The reports on decrease of breast cancer incidence in women 50-69 years old are related to improvement of preventive measurements such as mammography screening in developed countries. In the United States, Australia, and Western Europe this decrease seems to follow a decrease in hormonal therapy [30], as oestrogen plays important role in pathogenesis of breast cancer [31,32].

Factors involved in the development of breast cancer incidence include the socioeconomic status, some food additives, pesticides, oestrogen and progesterone replacement therapy, some antibiotics, radiation, mutations at genes BRCA1, BRCA2, metabolizing enzyme polymorphisms, epidermal growth factor and its receptor (HER), androgen levels, and insulin-like growth factor [33-35]. Age (including transplacental, prepubertal) may also play an important role in oestrogen exposure-related breast cancer risk that probably involves epigenetic mechanisms [36-39].

Currently there are some 160 xenoestrogens that may be involved in breast cancer development [40-42]. Women are the largest consumers of cosmetic products which may be a significant source of xenoestrogens. Some, such as metalloestrogens (e.g., aluminium salts), parabens, cyclosiloxanes, triclosan, UV screeners, phthalates, Aloe Vera extracts, and musk are present in numerous cosmetics products. Humans are exposed to these chemicals transcutaneously and measurable levels have been detected in human breast tissue [23].

Alcohol is also related with increased risk of breast cancer development as even low alcohol consumption increases serum oestradiol (especially for carriers of a certain alcohol dehydrogenase allele) and stimulates ER alpha [43,44]. On animal model it is shown that alcohol increases oestradiol levels in dams, which leads to higher levels of ER alpha receptors in their offspring mammary gland and may launch tumorigenesis [37].

The effect of diet on breast cancer development was observed in Japanese women after the Second World War when dramatic changes in their diet happened such as increased consumption of meat, eggs, and milk containing oestrogens or oestrogen-like compounds [45]. Milk is a source of oestrogen due to the practice of milking pregnant cows [46].

Heterocyclic amine and their metabolites, especially 2-amino-1-methyl6-phenylimidazo (4,5-b) pyridine (PhIP) is present in high concentrations in well-cooked meat and it binds to and activates the breast cell ER alpha [47,48]. In animal model, it causes breast cancers [49]. The suggested mechanisms of PhIP mechanisms are formation of PhIP-DNA adducts and increase of proliferation in mammary gland terminals and buds [50]. Similarly, at concentrations higher than 140 μg/m3 polyaromatic hydrocarbons from food may increase breast cancer risk in postmenopausal women if they were exposed to it early in life [51].

Styrene, a widely used plastic for food packing, has been associated with breast cancer risk both in men and women [52]. Direct intake of styrene is via food packed or even cooked in styrene boxes with a direct migration of styrene to food. Styrene and its metabolites bind to ER alpha [53], cross the placental barrier, and also affect the development of reproductive organs [54,55].

The carcinogenic potential of xenoestrogens may also depend on polymorphism of metabolic enzymes. It is shown that subpopulation carrying a polymorphism of metabolic enzyme CYP1A1 m2 is more susceptible to breast cancer development after exposure to polychlorinated biphenyls (PCB), which may explain contradictory epidemiological reports on the association between breast cancer incidence and PCB exposure [35,56,57].

## Lung cancer

Lung cancer is the predominant cause of cancer mortality [58]).There is a gender difference in the incidence of lung cancer types. The leading cause in men is the squamous cell carcinoma, and in women adenocarcinoma. Oestrogen and ER distribution could be the main cause of this difference [59,60]. Despite the fact that lung cancer incidence is increasing in women [61-64] studies reporting lung cancer incidence basically rarely give attention to possibly gender related susceptibility [65-67]. Lung cancer is about 70% ER beta positive. The ratio between ER alpha and ER beta in the lung tissue seems to be relevant for lung cancer development and may explain the higher incidence of lung adenocarcinoma in women than in men [68]. Increased lung cancer risk in women is associated with a lower social status and high level of indoor pollution with PCBs during cooking as a consequence of coal usage [69,70]. ER beta levels in lung cancer are gender related and have impact on survival rate [71]. While ER beta receptor positive or negative lung cancers has no impact on survival in women, in men ER beta positive lung cancer is associated with a significantly lower mortality than ER beta negative lung cancer [72]. ER alpha modulates lung differentiation and maturation while ER -beta causes proliferation of lung cancer cells [73,74]. Gender specific distribution of CYP19 (aromatase) in lung cells puts in correlation oestrogen levels and lung cancer etiology [75]. Additionally, women taking oestrogen therapy have shown increased lung cancer incidence [76]. Same as for breast cancer epidermal growth factor and its receptor HER plays a significant role in non-small cell carcinoma and is associated with endogenous estrogen exposure [77].

Smoking remains the major cause of lung cancer in humans [78]. Methylnitrosamino-pyridyl-butanone, a powerful carcinogenic agent contained in cigarette smoke is ER beta receptor related [69]. The activity of nicotine is gender-specific [79], since cotinine, a nicotine metabolite, is an aromatase inhibitor [80] that decreases oestrogen end increases testosterone levels. Polonium 210 in cigarettes [81] may have similar activity as other metalloestrogens [24,82]. Other carcinogens in cigarette smoke should be re-evaluated for their xenoestrogen or aromatase inhibitor potency.

Traffic air pollution is also related to lung cancer [83,84]. A number of compounds from traffic emissions are oestrogen ligand active compounds [85]. PAHs represent one of the major mixtures of agents that are present in polluted air have been demonstrated to affect oestrogen homeostasis [86-88].

Industrial emissions also contain pollutants with oestrogen-like activity, such as heavy metals and dioxins [89]. A significant association between industrial air pollutants and lung cancer risk has been reported in women [90].

**Figure 1:**
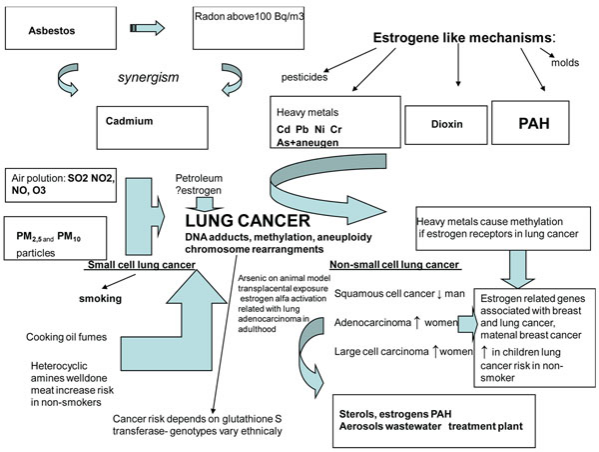
Schematic presentation of lung cancer development

Arsenic is a known lung carcinogen [6] whose biological effects include increased ER alpha transcription. In animals, transplacental exposure to arsenic causes lung cancer in female offspring. This suggests that arsenic can modify genes during foetal development which may cause lung cancer later in life [91]. Figure 1 shows complex environmental exposures which may lead to the lung carcinogenesis.

## Kidney cancer

Data on the environmental aetiology of kidney cancer are not available, and much more research is needed. The fact that renal cell carcinoma, the most common type of kidney cancer, can be induced by exposure to oestrogens [92] in animal model suggests the involvement of oestrogen receptors in the aetiology of kidney cancer and of a possible role of xenoestrogens. Kidney cancer incidence seems to be gender -related, with an incidence that is two times higher in men than in women [93]. In addition, genetic polymorphisms of ER alpha in the kidney seem to play a significant role in the development of kidney cancer [92]. Cadmium and arsenic as xenoestrogens may also induce kidney cancer [94,95]

## Pancreatic cancer

Understanding of pancreatic cancer aetiology is crucial, as it is the fourth leading cause of cancer deaths in the USA [64] and one of the most aggressive diseases. The incidence of pancreatic cancer has been relatively stable worldwide over the last few decades [96-98]. It is more frequent in men than in women [99,100]. Pancreatic cancer cells are ER alpha and beta positive and consequently may be modulated by oestrogen [101] which is consistent with similar mechanisms observed in xenoestrogen-related cancers [102,103]. There are very few data on the effects of xenoestrogens on pancreatic cancer incidence.

Methylnitrosamino-pyridyl-butanone is the only compound demonstrated to cause pancreatic cancer in animal models [104]. This finding has also been reported for the adenocarcinoma of the lung and has been related to ER beta activation [68].

It is also known that consumers of fried meat run a higher risk of pancreatic cancer development probably due to exposure to benzo(a)pyrene and other food contaminants that have xenoestrogenic properties [45,105].

## Brain tumour

Brain tumours are characterized by disturbances in ERs [106-108]. Transplacental exposure to xenoestrogens may increase the risk of brain tumour development [79,80]. Xenobiotics that inhibit aromatase also inhibit the conversion of oestrogen to testosterone and may have a significant impact on brain pathology, given the evidence that disturbed levels of testosterone have impact on apoptosis and intracellular signaling [109] Increased brain cancer incidence has been reported in humans living near petrochemical industries [110]. Despite the fact that the exact chemical composition of the mixture of air pollutants remains unknown, polycyclic aromatic hydrocarbons (PAH) are present in polluted air in such areas [67]. PAHs as xenoestrogen-like agents [87] may have played a causal role on the excess of brain cancer incidence detected

## Reproductive system

Testicular cancer incidence has significantly increased over the last few decades with yet no clear hypothesis on impact of environment on its aetiology [111]. As this trend cannot be explained by cryptorchidism, smoking, genetic variations, or physiological stress, the role of environmental exposure is being investigated to elucidate its aetiology and to identify preventive measures [112].

Both ER alpha and beta are expressed in the human testis and are involved in the control of testicular function [113]. The role played by xenoestrogens on testis development is still only partly known in the animal model, with results showing very dynamic changes in tissue sensitivity to xenoestrogens with unknown consequences during puberty and adulthood [114].

There are a limited number of studies reporting possible association between testicular cancer and disturbances in oestrogen levels. Testicular cancer has been reported in sons of smoking mothers [115], but also in mothers who were taking antiepileptics during pregnancy [116]. Both antiepileptics and cotinine from cigarette smoke are aromatase inhibitors. Transplacental exposure to aromatase inhibitors and consequently increased levels of testosterone may have long-term effects in humans, as shown in an animal model [117].

Epidemiological studies also suggest increased risk of testicular cancer following prenatal exposure to oestrogens [118].

Styrene storage containers may contaminate food which becomes a source of styrene exposure. Transplacental exposure to low levels of styrene may lead to the disturbed development of male genital organ [54]. However, its effect on cancer development remains unknown.

Endocrine disrupting chemicals which disturb ERs can cause female reproductive dysfunction [119,120]. As ovarian cancer therapy is still not marked with significant success and mortality is very high it is of major significance elucidation of its aetiology [121].

Cadmium, one of most investigated heavy metals, has a significant role on ovarian and reproductive functions, as it lowers progesterone levels and mimics oestrogen in various tissues by binding to ER alpha. Cadmium is not confined to occupational exposure alone; it is found in cigarette smoke, food, nickel/cadmium batteries, pigments, and plastics [122].

Ovarian cancer is associated with milk and cheese consumption due to oestrogen and insulin growth factor present in milk of pregnant cows [46,123].

According to recent experimental evidence, uranium in water should be further considered in research as an additional risk for reproductive cancers [82].

## Conclusions

As an evolutionary conserved molecule, oestrogen is present in both plants and animals. Oestrogen is recognized today as a critical modulator of development, homeostasis in adulthood and orchestration of response to environment.

Although gene polymorphisms can change cancer incidence [124,125], it is clear that environment has predominance over genes in cancer risk. Responses to environmental stressors are age- and gender related, and transplacental exposure to xenoestrogens has been shown to have long-term effects in experimental models, as it modulates hormonal response in puberty This means that exposure to endocrine agents not only poses a health risk during exposure, but also increases susceptibility later in life [114,126,127]. Differences in susceptibility to xenoestrogens may be related to steroid and xenobiotic receptor levels, which are high in young adults (15-38y old) and decrease with age [128].

Current estimates of cancer risk in humans do not account properly for transplacental and environmental (including occupational) exposure to xenoestrogens. The role of xenoestrogens in cancer development should be re-evaluated using a new approach that would reflect the complexity of carcinogenesis.

Reductionism as the main scientific philosophy of the 20^th^ century gave a significant input to environmental health. However, the quantity of data available in noosphere, systems biology as a tool and new softwares for data sharing enable the investigation of interactions between xenoestrogens and other environmental stressors, such as radiation, and add new dimensions to the research of cancer aetiology using complexity as a new scientific philosophy. Contemporary mathematical models and systems biology allows the incorporation of all available data and modeling cancer risk allowing free interaction and clustering of data.

The collaboration of environmental health with oncology would be of crucial significance in the study of xenoestrogens as estrogen has today significant position in oncological diagnostics and therapy what includes measurements of ER levels in different tissues. Clinical oncology today takes part in scientific projects in order to achieve optimal treatment at the individual level (tailored therapy) and produces large amounts of data that reflect a tight gene-environment interaction and point to age and gender specific susceptibility. Collaboration between pharmacokineticians, oncologists, histopathologists, molecular epidemiologists, and genotoxicologists may improve our knowledge of cancer aetiology and lead to gender specific and final individualized therapies.

Available information systems and building of integrated exposure-disease pathways will give policymakers much more useful input in future for more efficient regulations than a large number of agent- and disease-specific studies.

## Competing interests

There is no competing interest in interpretation of data or presentation of information in this article which may be influenced by personal or financial relationship with other people or organizations.

## Authors' contributions

AF gave the basic idea/hypothesis and drafted the manuscript. MG, ZF and JK participated in its design and helped to draft the manuscript. MAK and AB gave significant contribution in modelling of approach of data interpretation. DFM drafted the manuscript and gave significant contribution in interpretation of collected data. All authors read and approved the final manuscript.
